# *Pinus sylvestris* Essential Oil-Loaded Gelatin–Chitosan–Snail Slime Nanofibrous Mats for Active Food Packaging Applications

**DOI:** 10.3390/polym18131648

**Published:** 2026-07-02

**Authors:** Ghizlane Akhouy, Salih Birhanu Ahmed, Cemhan Dogan, Mehmet Durmus Calisir, Manal Zefzoufi, Faissal Aziz, Nagham Elberishy, Yasin Akgul, Islam Shyha

**Affiliations:** 1 Water Sciences, Microbial Biotechnologies and Natural Resources Sustainability Laboratory, Faculty of Sciences Semlalia, Cadi Ayyad University, Marrakech 40000, Morocco; ghizlaneakhouy86@gmail.com (G.A.); f.aziz@uca.ma (F.A.); 2 National Center for Research and Studies on Water and Energy, Cadi Ayyad University, Marrakech 40000, Morocco; 3 School of Mechanical and Industrial Engineering, College of Technology and Built Environment, Addis Ababa University, Addis Ababa 1000, Ethiopia; salihbirhanu@gmail.com; 4 Department of Food Technology, Bogazliyan Vocational School, Yozgat Bozok University, 66100 Yozgat, Türkiye; cemhan.dogan@yobu.edu.tr; 5 Faculty of Engineering and Architecture, Recep Tayyip Erdogan University, 53200 Rize, Türkiye; mehmetdcalisir@gmail.com; 6 Bioorganic Chemistry Team, Faculty of Science, Chouaïb Doukkali University, El Jadida 24000, Morocco; zefzoufi.manal1994@gmail.com; 7 Production Engineering Department, Faculty of Engineering, Alexandria University, Alexandria 21544, Egypt; 8 Department of Industrial Engineering, University of Business and Technology, Jeddah 23847, Saudi Arabia; 9 Department of Biomedical Engineering, Faculty of Engineering and Natural Sciences, Karabuk University, 78050 Karabuk, Türkiye; 10 School of Computing Engineering and the Built Environment, Edinburgh Napier University, Edinburgh EH10 5DT, UK

**Keywords:** biodegradable, bioactivity, food packaging, polymer, solution blow spinning, nanofibrous

## Abstract

Developing biodegradable and functional polymeric materials for active food packaging is essential to mitigate the environmental burden of petroleum-based plastics. In this context, gelatin/chitosan (G–Ch) nanofibrous mats were fabricated via solution blow spinning (SBS) and functionalized with snail slime (SS) and *Pinus sylvestris* essential oil (PSEO) to enhance their bioactivity and barrier performance. SS is rich in glycoproteins and natural bioactive compounds, while PSEO is characterized by terpene-based antimicrobial and antioxidant activities. SS and PSEO were incorporated into the G–Ch polymeric matrix to enhance the bioactivity, structural functionality and preservation performance of the nanofibrous mats. Three formulations (G–Ch, G–Ch–SS, and G–Ch–SS–10PSEO) were designed to elucidate the influence of snail slime and essential oil incorporation on the structure–property–function relationships of the nanofibrous mats. Morphological analysis revealed a smooth and bead-free fibrous structure across all formulations. The average fiber diameter (AFD) increased from 191.83 nm for G–Ch to 263.88 nm for G–Ch–SS and 295.83 nm for G–Ch–SS–10PSEO. FTIR and XRD analyses showed the physical encapsulation of the active compounds without significant chemical interactions. Furthermore, the incorporation of PSEO increased surface hydrophobicity and reduced air permeability, indicating the formation of a more compact fibrous structure with enhanced barrier properties. The functional performance of the nanofibrous mats was significantly improved by the addition of snail slime and PSEO. The G–Ch–SS–10PSEO formulation exhibited the highest antioxidant activity, reaching 36.8% for DPPH and 42.7% for ABTS, along with enhanced antibacterial efficacy against both *Escherichia coli* (*E. coli*) and *Staphylococcus aureus* (*S. aureus*). Application tests on chicken wings demonstrated that the bioactive nanofibers effectively suppressed microbial growth, limited pH increases, and reduced lipid oxidation during 14 days of refrigerated storage. Overall, the results demonstrate that the synergistic integration of snail slime and essential oil within a biodegradable polymer matrix provides a promising strategy for designing active nanofibrous materials with enhanced structural and bioactive properties for sustainable food-packaging applications.

## 1. Introduction

The widespread use of petroleum-based plastics in food packaging has resulted in significant environmental challenges due to their persistence and resistance to degradation [1]. The scale of plastic consumption is substantial, with approximately 500 billion plastic bags used annually worldwide, contributing to the accumulation of plastic waste in natural ecosystems. As a result, recent estimates indicate that more than 5.25 trillion micro- and nanoplastic particles are present in marine environments [2]. Beyond environmental contamination, these pollutants pose direct risks to human health; notably, microplastic residues have been detected in one out of every three fish consumed by humans [3]. Consequently, there is an increasing demand for the development of biodegradable and environmentally friendly polymeric materials that can replace conventional packaging systems while maintaining functional performance such as antimicrobial activity, antioxidant capacity, barrier properties, and food preservation efficiency.

These alarming figures highlight the urgent need to develop sustainable, biodegradable, and environmentally responsible alternatives for food-packaging applications.

Biodegradable polymers have attracted considerable attention as a promising alternative for sustainable packaging due to their ability to decompose into environmentally benign by-products such as carbon dioxide, water, and biomass, thereby supporting a circular materials economy [4]. Protein–polysaccharide blends are increasingly considered promising matrices for biodegradable and active food packaging because they can combine the complementary properties of both biopolymer classes. Their association may produce a denser and more cohesive network through multiple intermolecular interactions between the functional groups of polysaccharide chains and the amino acid residues of proteins. This results in improved mechanical integrity compared with the individual components. Protein incorporation can also decrease water solubility and improve the water resistance and stability of polysaccharide-based materials. In addition, when plant-derived polysaccharides or agro-industrial residues are used, naturally occurring polyphenols may impart antioxidant, antimicrobial, and UV-protective properties [5]. Gelatin is one of these materials, and it is widely used in nanofiber fabrication owing to its excellent spinnability, biocompatibility, biodegradability and low cost [6]. Chitosan, another well-known biopolymer, offers inherent antimicrobial activity and can enhance the mechanical properties of gelatin-based systems when incorporated as a reinforcing component. Previous studies have reported that gelatin–chitosan exhibit improved antibacterial performance against both *E. coli* and *S. aureus* [7]. Similarly, recent findings have demonstrated that the incorporation of chitosan into gelatin significantly improved the mechanical strength of the composites [8].

Snail slime (SS), derived from *Helix aspersa* Müller, has recently gained attention as a multifunctional biomaterial due to its rich composition of glycoproteins, polyphenols, and natural antioxidants, which contribute to its antimicrobial and antioxidant properties [9,10]. Essential oils such as *Pinus sylvestris* essential oil (PSEO) are well known for their strong bioactivity, primarily attributed to terpene-based compounds, including α-pinene and β-pinene species, which exhibit significant antimicrobial and radical-scavenging effects [11], making PSEO a promising natural additive for functional food-packaging systems [12]. A rational strategy to design multifunctional nanofibrous mats involves a stepwise functionalization approach. Initially, the incorporation of bioactive components such as snail slime into gelatin–chitosan matrices enhances bioactivity while modifying the structural characteristics of the nanofibrous mats. Subsequently, the integration of essential oils further improves the antimicrobial and antioxidant performance, resulting in a synergistically enhanced multifunctional material with improved structure–property–function relationships. In this context, the combined incorporation of snail slime and PSEO is particularly relevant because of their distinct physicochemical nature and complementary functional roles. Snail slime is predominantly hydrophilic and contains water-soluble bioactive constituents, whereas PSEO is a hydrophobic and volatile mixture rich in lipophilic compounds. Their incorporation into G-Ch nanofibrous mat enables the coexistence of bioactive fractions with different polarity and solubility. Snail slime contributes hydrophilic biological components, while PSEO provides antioxidant and antibacterial compounds, thereby broadening the multifunctional potential of the developed nanofibrous mat.

Polymer nanofibers have attracted considerable attention in advanced packaging applications due to their high surface area, interconnected porous structure, and tunable morphology, which promote efficient mass transfer, enhanced surface interactions, and the incorporation of active functional agents. These structural characteristics can be tailored to achieve the desired balance between permeability and barrier performance depending on the packaging application. These structural features allow nanofibrous mats to enhance the retention and distribution of active compounds, thereby improving the functional performance of packaging materials, particularly in terms of antimicrobial activity and shelf-life extension [13]. Among the available fabrication techniques, solution blow spinning (SBS) has emerged as a scalable and efficient method for nanofiber production. Beyond their distinct fiber-forming mechanisms, electrospinning and solution blowing also differ in the quality of the resulting nanofibrous mats. Electrospinning typically yields fibers with narrower diameter distribution, greater uniformity, and fewer structural defects, which can improve mechanical consistency and barrier properties. In contrast, solution blowing provides higher production throughput and better industrial scalability but often produces mats with higher porosity, wider fiber diameter distribution, and less uniform thickness [14]. These structural differences can significantly influence the final functional properties of the mats, including permeability, active compound release, and mechanical performance. Unlike electrospinning, which relies on high-voltage electric fields, SBS utilizes high-velocity air streams to generate fibers, offering advantages such as higher production rates, simpler processing and improved industrial applicability [15,16].

Gelatin–chitosan nanofibrous mats have attracted interest as carriers for natural bioactive compounds in food-packaging applications. Previous studies have extensively explored gelatin- and chitosan-based nanofibrous systems incorporating various essential oils and bioactive compounds for active food-packaging applications. For instance, Elomar et al. [17] developed gelatin/chitosan nanofibers functionalized with eucalyptus essential oil using the electroblowing technique and reported enhanced antibacterial activity, thermal stability, and air permeability. Similarly, Tang et al. [18] fabricated electrospun gelatin nanofibers loaded with peppermint and chamomile essential oils, demonstrating improved antioxidant activity and UV protection. In another study, Zhou et al. [19] incorporated angelica essential oil into gelatin nanofibers and observed enhanced hydrophobicity, antioxidant, and antimicrobial performance. Furthermore, Duan et al. [20] reported electrospun gelatin/chitosan nanofibers containing curcumin for active and intelligent packaging, showing pH-responsive and antioxidant properties. These studies demonstrate the versatility of gelatin/chitosan nanofibrous systems as carriers for functional bioactive agents. However, to the best of our knowledge, the incorporation of snail slime as a multifunctional natural additive in combination with *Pinus sylvestris* essential oil within a gelatin/chitosan nanofibrous matrix has not been previously reported for active food-packaging applications, highlighting the novelty of the present study. It has generally evaluated their morphological, physicochemical, antioxidant, and antimicrobial properties without direct validation on food products. Snail slime, meanwhile, has been investigated in biomedical and other functional material applications rather than in nanofibrous food-packaging materials. Consequently, studies simultaneously integrating gelatin, chitosan, snail slime, and PSEO into a nanofibrous mat for food preservation remain scarce. This gap supports the relevance of the present work, in which the developed bioactive mat was evaluated for chicken wings packaging.

In this study, G-Ch-based nanofibrous mats were designed through a stepwise functionalization strategy by first incorporating SS and subsequently integrating PSEO to develop a multifunctional bioactive system. Unlike conventional approaches, this design enables the progressive enhancement of structural and functional properties through controlled incorporation of bioactive components. Although previous studies have explored essential oil-loaded nanofibers or snail-slime-based systems separately, their combined integration within a nanofibrous matrix remains largely unexplored. The novelty of this study lies in the development and comprehensive evaluation of gelatin–chitosan nanofibrous mats incorporating snail slime and PSEO for active food-packaging applications. The effects of these bioactive components were investigated through morphological, structural, and physicochemical analyses, water contact angle, and air permeability measurements, as well as antioxidant and antibacterial assessments. Therefore, this work aims to investigate the influence of this multicomponent on the morphology, surface characteristics, barrier properties and bioactive performance of the nanofibers, while also evaluating their applicability in food packaging. This approach provides new insights into the design of multifunctional, biodegradable nanofibrous mats with enhanced structure–property–function relationships for active food-packaging applications.

## 2. Materials and Methods

### 2.1. Materials

The chitosan was a medium molecular weight grade with a degree of deacetylation of 75–85%, a molecular weight range of approximately 190–310 kDa, and a viscosity range of 200–800 cP purchased from Labor-Teknik Laboratory Supplies Industry and Trade Inc., (Istanbul, Turkiye). The material was supplied as an off-white powder and was readily soluble in dilute acetic acid for solution preparation. Gelatine 250–270, type B, Bloom Edible, produced from bovine skin, was supplied in powder form from Halavet Gida LLC. (Istanbul, Türkiye). Formic acid (85%, 1.20 g/cm^3^) and acetic acid (80% purity, 1.08 g/cm^3^) were supplied by Tekkim (Bursa, Türkiye). All chemicals were used without further purification.

*Pinus sylvestris* was collected from the Oukaimeden region, Morocco. The fresh fruits were washed, dried in the shade at room temperature, and then ground into a fine powder. A reference specimen was deposited in the herbarium of the Department of Botany at the Scientific Institute of Rabat, Morocco.

The essential oil of *Pinus sylvestris* (PSEO) was extracted by hydrodistillation of the powdered *Pinus sylvestris* fruit using a Clevenger-type apparatus for 4 h. Anhydrous sodium sulfate was then used to dry the collected essential oil. The dried PLEO was finally stored in airtight containers at 4 °C, protected from light, until use.

The adult (12–15 months) *Helix aspersa* snails were supplied by the International Academy of Heliciculture of Marrakech (AIHM). Snail slime was collected using a non-invasive and non-destructive procedure under hygienic conditions. No harmful treatment, invasive manipulation, or sacrifice was involved during the collection process, and the snails were returned to their normal living conditions after mucus extraction.

The snails were washed under running tap water and then placed in an extraction chamber provided by AIHM. The extraction was performed for 1 h using water spraying without any chemical stimulants, and the process was repeated three times. Afterwards, the slime collected was transferred to a different container after an adequate microfiltration (100 µm) for the purification of the residue. A dry powder was produced by subjecting the obtained liquid to freeze-drying through lyophilization over a period of 48 h.

### 2.2. Fabrication Methods

Table 1 shows the composition, solvent system, and preparation conditions of the three solutions prepared for SBS.

The proportions of the individual components were selected based on literature-reported formulations, component compatibility, and the targeted properties of active food-packaging nanofibrous mats. The relatively high polymer content was intended to promote continuous fibers with sufficient diameter and to produce a coherent nanofibrous mat with adequate structural integrity for handling and packaging applications. Gelatin served as the main fiber-forming matrix, while chitosan contributed to matrix cohesion and functional performance. Snail slime and PSEO were incorporated as bioactive components to enhance antioxidant and antimicrobial properties without dominating the polymer phase or preventing fiber formation.

**Figure 1:**
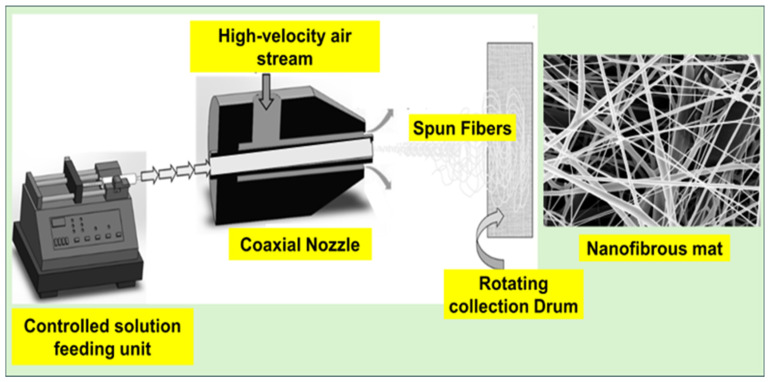
Schematic diagram of the used system.

Nanofibrous mats were produced using the SBS system, as shown in Figure 1. The system has compressed air operating at 1 bar to generate high-velocity airflow for fiber formation. The solution was delivered using a syringe pump with a flow rate of 10 mL/h via a nozzle needle. The resulting fibers were collected on a rotating collector located 45 cm from the nozzle tip and deposited onto a spun-bond polypropylene nonwoven substrate. After fabrication, the nanofibrous mats were carefully peeled off and separated from the PP substrate before further characterization and food preservation experiments, ensuring that the measured properties were solely attributed to the fabricated gelatin/chitosan-based nanofibrous mats.

To improve the water resistance and structural stability of the gelatin–chitosan-based nanofibrous mats, the as-prepared samples were thermally treated at 120 °C for 2 h under ambient conditions. This post-treatment was also intended to facilitate residual solvent removal. The spinning process was carried out for three hours under optimized solution blow spinning conditions until a uniform nanofibrous mat was obtained. The nozzle used had an inner diameter of 0.51 mm (21 G).

### 2.3. Characterization Methods

The chemical composition of PSEO was analyzed using a Thermo 8000 GC–MS system (Thermo Fisher Scientific, Waltham, MA, USA) equipped with an RTX-5 capillary column (30 m × 0.32 mm, 0.25 µm film thickness). The oven temperature was initially set at 40 °C for 6 min, then increased at a rate of 180 °C and held for 10 min. Helium was used as the carrier gas. The injector and detector temperatures were maintained at 225 °C and 240 °C, respectively. Compounds were identified by comparing their mass spectra with standard libraries.

The chemical characterization of SS is performed using two complementary Liquid Chromatography–Tandem Mass Spectrometry (LC–MS/MS) methods. The first method targets amino acids, peptides, and collagen-derived molecules, enabling an accurate assessment of the proteinaceous fraction of the slime. The second method focuses on bioactive secondary metabolites such as phenolic compounds, flavonoids, and vitamins. In addition, the concentrations of key components were determined using quantitative analysis, allowing the results to be expressed in mg/g or mg/kg units. This combined analytical approach provides both qualitative identification and quantitative evaluation of the bioactive composition of snail slime.

The morphologies of nanofibrous mats were investigated using a Carl Zeiss Ultra Plus Field Emission Scanning Electron Microscope (FE-SEM) (Carl Zeiss AG, Oberkochen, Germany). Fiber diameter distributions were determined from SEM images using ImageJ software (version 1.53e), based on 100 measurements taken from randomly selected regions.

The Fourier-transformed Infrared (FTIR) spectra of the samples were recorded using a Bruker ALPHA FTIR Spectrometer (Bruker, Billerica, MA, USA) in the range of 400–4000 cm^−1^ with 24 scans.

Surface wettability was determined using an optical tensiometer (Theta Lite, Biolin Scientific, Gothenburg, Sweden) via the sessile-drop method with a droplet volume of 0.0085 mL. Each sample was tested at three different locations, and the results are reported as mean ± standard deviation (*n* = 3).

The air permeability of the fibrous mats was assessed using the Prowhite Air Test II device in accordance with ASTM D737 standards [21]. The test used a 38 cm^2^ sample size and an air pressure of 125 Pa. The experiment was performed at room temperature. All data are expressed as mean ± standard error with three replicates (*n* = 3).

The tensile properties of all samples were determined using uniaxial tensile testing with a crosshead speed of 1 mm/min. All samples were cut into rectangles with dimensions of 100 × 20 mm^2^ and horizontally mounted on gripping units, leaving a 40 mm gauge length.

XRD analysis was performed using a RIGAKU ULTRA IV XRD diffractometer (Rigaku, Tokyo, Japan) over a 2θ range of 10–90° at room temperature. The X-ray source was operated at 30 kV and 20 mA, with a step size of 0.03°.

The swelling behavior of the nanofibrous mats was evaluated to determine their water sensitivity. Samples were cut into rectangular pieces of 20 × 20 mm and dried at 40 °C until constant weight. The initial dry weight of each sample was recorded as W_0_. The dried samples were then immersed in distilled water at room temperature for 1 h. After immersion, the samples were carefully removed, and excess surface water was gently blotted using filter paper without compressing the mat structure. The swollen weight was recorded as W_s_. The swelling ratio was calculated using the following equation:

Swelling ratio (%) = [(W_s_ − W_0_)/W_0_] × 100

Moisture uptake was determined by placing pre-dried nanofibrous mat samples in a desiccator containing saturated NaCl solution to maintain a relative humidity of approximately 75% at 25 °C. After 24 h, the samples were weighed again, and the moisture-equilibrated weight was recorded as W_m_. Moisture uptake was calculated according to the following equation:

Moisture uptake (%) = [(W_m_ − W_0_)/W_0_] × 100

The amount of PSEO retained in the nanofibrous mats after thermal treatment was determined using a UV–Vis spectrophotometric method. Briefly, accurately weighed G–Ch–SS–10PSEO nanofibrous mat samples before and after thermal treatment were cut into small pieces and immersed in n-hexane/ethanol solution (90:10, *v*/*v*) at a solid-to-solvent ratio of 10 mg/mL. The mixtures were sonicated for 20 min and then shaken at room temperature for 1 h in the dark to extract the PSEO from the nanofibrous matrix. The extracts were centrifuged at 5000 rpm for 10 min, and the supernatants were collected for spectrophotometric analysis.

A calibration curve was prepared using known concentrations of pure PSEO in the same solvent system. The absorbance was measured at the maximum absorption wavelength of PSEO determined by spectral scanning between 200 and 400 nm. Blank extracts obtained from G–Ch–SS nanofibrous mats without PSEO were used for baseline correction to eliminate possible interference from the polymer matrix and snail slime components. The PSEO content was expressed as mg PSEO equivalent per g of dry nanofibrous mat. PSEO retention after thermal treatment was calculated using the following equation:

PSEO retention (%) = (PSEO content after thermal treatment/PSEO content before thermal treatment) × 100

The antioxidant activity of the nanofiber samples was determined using DPPH and ABTS radical scavenging assays. The specimens (approximately 50 mg) were extracted in 10 mL of 80% ethanol (*v*/*v*) for 1 h at room temperature (200 rpm, protected from light), and the extracts were filtered through a 0.45 µm membrane. For the DPPH assay, 1.5 mL of the extract was mixed with 1.5 mL of freshly prepared DPPH solution (0.1 mM in methanol), vortexed briefly, and incubated for 30 min in the dark. The absorbance was measured at 517 nm using an Optizen UV–VIS spectrophotometer (Mecasys, Daejeon, Republic of Korea). For the ABTS assay, ABTS•^+^ was generated by reacting 7.0 mM ABTS with 2.45 mM potassium persulfate (1:1, *v*/*v*) and allowing the solution to stand in the dark for 12–16 h. The resulting radical cation solution was diluted with 80% ethanol to an absorbance of 0.70 ± 0.02 at 734 nm. Then, 2.0 mL of the ABTS•^+^ working solution was mixed with 200 µL of the extract and incubated for 6 min at room temperature before measuring the absorbance at 734 nm. The radical scavenging effect (%) for both assays was calculated according to Equation (1):

(1) $$Radical\;scavenging\;\%\;=\left(\frac{A_{0}-A_{s}}{A_{0}}\right)\times 100$$

where A_0_ represents the absorbance of the control (without sample) and A represents the absorbance in the presence of the extract. All measurements were conducted in triplicate, and the results were expressed as mean ± standard deviation.

The antibacterial efficacy of the nanofibrous mats was examined against *Escherichia coli* (*E. coli*) and *Staphylococcus aureus* (*S. aureus*) using a disk diffusion method. Samples were cut into disks (6 mm diameter) and sterilized by UV irradiation for 15 min on each side immediately prior to testing. Overnight bacterial cultures were diluted to approximately 10^6^ CFU/mL (equivalent to 0.5 McFarland) and evenly spread onto Mueller–Hinton agar plates. The nanofiber disks were then placed on the agar and incubated at 37 °C for 24 h. After incubation, the total diameter of the inhibition area was measured using a digital caliper. Since the nanofiber disks had a diameter of 6 mm, the antibacterial results were expressed as the net clear inhibition zone after subtracting the disk diameter from the total measured diameter, according to the following equation:

Net inhibition zone (mm) = total inhibition diameter (mm) − 6 mm

All experiments were performed in triplicate, and the results were reported as mean ± standard deviation. The antibacterial performance was comparatively evaluated among different formulations.

### 2.4. Application of Nanofibrous Mats and Analyses

**Figure 2:**
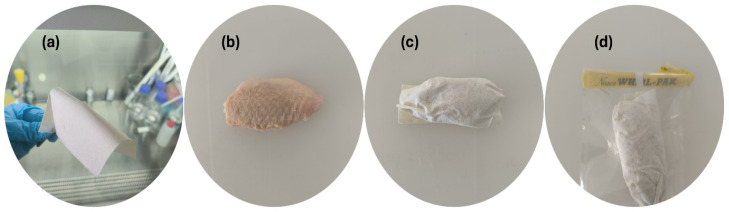
Application steps of the snail slime-based nanofibrous mats on chicken wings. (**a**) The gelatin/chitosan–snail slime nanofibrous mat (G–Ch–SS–10PSEO) after UV sterilization. (**b**) Fresh chicken wing sample prior to packaging. (**c**) Chicken wings wrapped completely with the nanofiber mat to ensure full surface coverage. (**d**) Packaged sample placed into a sterile Whirl-Pak^®^ bag, Whirl-Pak (Pleasant Prairie, WI, USA) for refrigerated storage at 4 ± 1 °C until analysis.

Fresh chicken wings were used to evaluate the preservation performance of the nanofibrous mats under natural spoilage conditions (Figure 2). Wings were portioned into uniform pieces (approximately 25–30 g) and individually placed in sterile polyethylene bags. Before application, the nanofiber mats were sterilized under UV light for 15 min on each side. Each chicken piece was then wrapped with a single layer of the sterilized nanofibers (G–Ch, G–Ch–SS, or G–Ch–SS–10PSEO), ensuring complete surface contact, while unwrapped wings served as controls. All samples were stored at 4 ± 1 °C, and analyses were performed on days 0, 3, 7, and 14.

For microbiological evaluation, 10 g of each sample was aseptically homogenized in 90 mL sterile peptone water, serially diluted, and plated onto Plate Count Agar to determine total viable counts (TVC). The plates were incubated at 37 °C for 48 h, and the results were expressed as log CFU/g. The pH of chicken samples was determined by homogenizing 10 g of meat with 100 mL of distilled water and measuring the slurry using a calibrated digital pH meter at room temperature. Lipid oxidation was assessed using the thiobarbituric acid reactive substances (TBARS) assay. Briefly, 5 g of sample was homogenized with trichloroacetic acid, filtered, and reacted with thiobarbituric acid at 95 °C. The absorbance was measured at 532 nm, and the results were expressed as mg malondialdehyde (MDA) per kg of sample. All measurements were performed in triplicate.

## 3. Results and Discussion

### 3.1. Chemical Composition of Bioactive Components

The chemical composition of *Pinus sylvestris* essential oil (PSEO) is presented in Table 2. A total of 16 compounds were identified, accounting for 99.75% of the total oil content. The composition was dominated by monoterpenes, with 3-carene (30.14%), α-pinene (20.11%), myrcene (10.70%), β-pinene (8.70%), and caryophyllene (8.90%) as the major constituents. The presence of bioactive terpenes such as α-Pinene and β-Pinene highlights the potential of PSEO in inhibiting microbial growth, which is critical for extending product shelf life [17]. Minor components such as α-Cadinene, α-Humulene, Verbenone, and α-Limonene were also detected, which may further contribute to the overall bioactivity through synergistic interactions. Overall, the high terpenoid content of PSEO, together with synergistic effects among its constituents, supports its use as a functional additive in nanofibrous mats, contributing to enhanced antimicrobial and antioxidant performance.

The composition of SS, presented in Table 3, reveals a complex mixture of organic and inorganic components. The organic fraction is rich in bioactive compounds, including allantoin (6.05 mg/g), glycolic acid (8.15 mg/g), collagen (489.5 mg/g), polyphenols (105.12 mg/g), and flavonoids (56.12 mg/g), along with essential vitamins (A, B1, E, and C). These constituents are known to contribute to the antioxidant and antimicrobial properties of SS. The mineral profile of SS includes zinc (10 mg/kg), magnesium (8 mg/kg), and potassium (6 mg/kg). Among these elements, zinc is particularly recognized for its antibacterial activity [22]. The diverse composition of SS highlights its potential as a multifunctional bioactive component, which may contribute to enhancing functional performance when incorporated into nanofibrous mats.

### 3.2. Morphologies of Nanofibrous Mats

**Figure 3:**
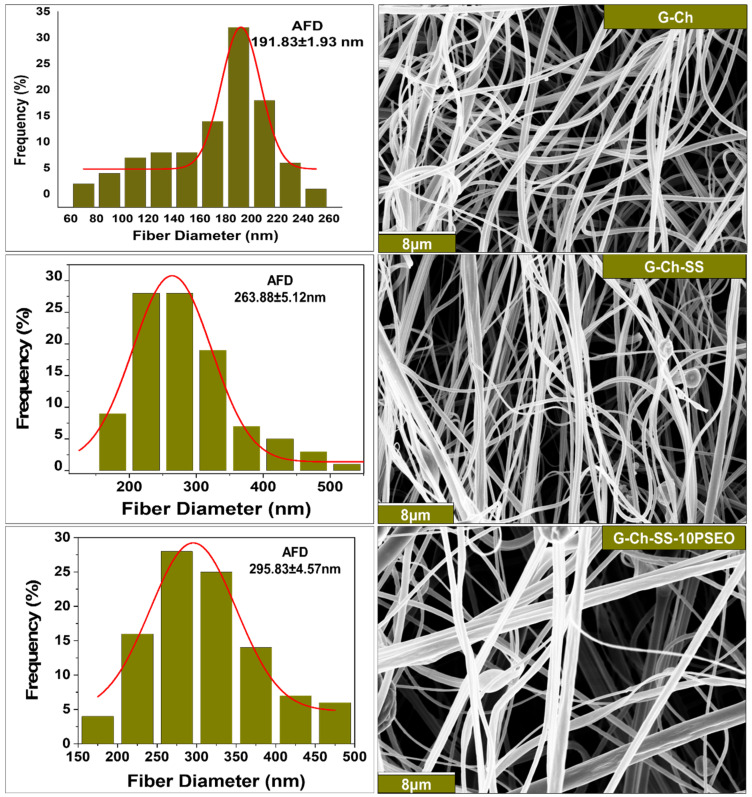
SEM micrographs of nanofibrous mats and their fiber diameter distributions.

The surface morphology and average fiber diameter (AFD) distribution of nanofibrous mats are presented in Figure 3. SEM images revealed uniform, smooth, and bead-free fibers for all formulations (G–Ch, G–Ch–SS, and G–Ch–SS-10PSEO), indicating stable fiber formation during the SBS process. A progressive increase in AFD was observed with the incorporation of SS and PSEO. The AFD increases from 191.83 ± 1.93 nm for G–Ch fibers to 263.88 ± 5.12 nm for G–Ch–SS fibers, and further to 295.83 ± 4.57 nm for the G–Ch–SS-10PSEO. This increase can be attributed to changes in solution properties, particularly an increase in viscosity and the presence of additional molecular interactions introduced by SS and PSEO, which affect jet stretching and thinning behavior during SBS. Similar trends have been reported in the literature, where the incorporation of essential oils, such as Origanum elongatum, into polymer solutions resulted in increased fiber diameter during fiber production [8]. And also, the average thickness of the deposited nanofibrous mats was measured using a digital micrometer and determined to be 717 ± 50.81 µm, 763 ± 59.10 µm, and 848.8 ± 68.72 µm for the G–Ch, G–Ch–SS, and G–Ch–SS-10PSEO samples, respectively. In addition, the fiber diameter distribution became, with the incorporation of bioactive components, indicating decreased homogeneity compared to G–CH nanofibers. This behavior may be associated with the complex composition of SS, which includes various organic and mineral constituents that can disrupt uniform polymer chain interactions and influence jet stability during fiber formation.

### 3.3. Structural Characterization of Nanofibrous Mats

**Figure 4:**
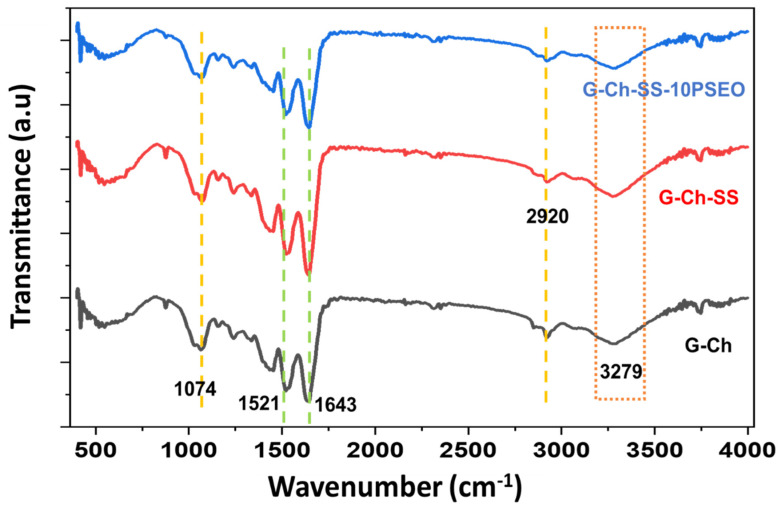
FTIR analysis of nanofiber mats.

FTIR spectroscopy was performed to investigate the chemical interactions and potential interactions among G–Ch, SS and PSEO. The spectra are presented in Figure 4. The FTIR spectra of G–Ch display characteristic peaks at 3279 cm^−1^, 1074 cm^−1^, and 1643 cm^−1^ corresponding to the stretching vibration of O–H, –C–O–C– bonds, and amid I, respectively [17]. The peak observed at 1536 cm^−1^ is attributed to amide II [23], while the band at 2941 cm^−1^ corresponds to C–H stretching vibration [8]. According to the literature, *Helix aspersa* slime exhibits a broad O–H and N–H band near 3286 cm^−1^, an amide I band around 1639 cm^−1^, and additional carbonyl, carboxylate, and glycosidic vibrations in the regions near 1740, 1400, 1366, and 1212 cm^−1^. In the G–Ch–SS nanofibrous mats, however, no separate bands could be attributed exclusively to SS. This is likely because the main functional groups of SS, particularly O–H, N–H, C=O, and C–O-containing groups, absorb in spectral regions already dominated by the G–Ch matrix [24].

Comparison of the FTIR spectra of G–Ch, G–Ch–SS, and G–Ch–SS-10PSEO samples revealed no significant peak shifts or appearance of new bands. This observation suggests that the incorporation of SS and PSEO did not induce major chemical modifications in the polymer matrix. Instead, the interactions between the components are likely dominated by physical interactions such as hydrogen bonding and intermolecular forces. Accordingly, the bioactive agents are likely dispersed within the nanofibrous mats. These findings are consistent with previous studies reporting that the incorporation of essential oils into biopolymer matrices does not significantly alter FTIR spectra [25]. This behavior is favorable for preserving the intrinsic chemical structure of the polymers while enabling the incorporation of bioactive compounds.

**Figure 5:**
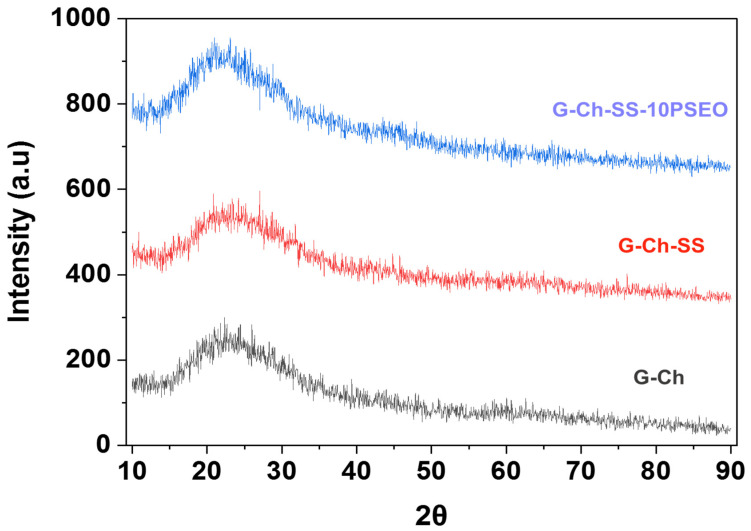
X-ray diffraction patterns of nanofibrous mats.

The XRD patterns of the nanofibrous samples are shown in Figure 5. All samples exhibited a broad halo peak in the range of 2θ = 16–30°, indicating the predominantly amorphous nature of the gelatin–chitosan matrix, which is in agreement with previous work [20]. The addition of SS and PSEO to the G–Ch nanofibers did not result in significant changes in the XRD patterns, suggesting that no major structural reorganization or crystallization occurred upon incorporation of the bioactive components. This observation further supports that SS and PSEO are dispersed within the polymer matrix without altering its overall structural organization.

### 3.4. Surface Wettability and Barrier Performance

**Figure 6:**
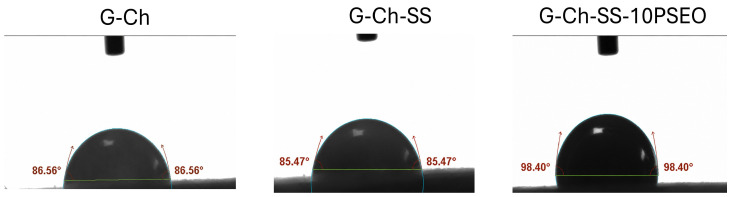
Images of water contact angle measurement for all samples.

The water contact angle (WCA) measurements were conducted to evaluate the surface wettability of the nanofibrous mats, and the results are presented in Table 4. The G-Ch nanofibers exhibited an average water contact angle of 86.56 ± 11.60°, indicating a moderately hydrophilic surface. The incorporation of SS resulted in a slight decrease in the contact angle to 85.47 ± 3.22°, suggesting that SS does not affect the surface wettability of the matrix. In contrast, the addition of 10 wt% of PSEO increased the contact angle to 95.40 ± 17.04°, indicating a transition toward a more hydrophobic surface. This behavior can be attributed to the intrinsic hydrophobic nature of essential oils, which tend to reduce surface energy and increase water repellency [19]. The increased hydrophobicity is beneficial for food-packaging applications, as it may enhance resistance to moisture penetration. The representative image of the water contact angle (WCA) measurement is shown in Figure 6.

Nanofiber-based food packaging differs from conventional paper or film-based systems due to its inherently porous structure, which enables controlled air circulation and can contribute to maintaining food freshness [8]. The air permeability values of the produced nanofibrous mats are presented in Table 4, showing 20.66, 13.00, and 14.66 mm/s for G–C, h, G–Ch–SS, and G–Ch–SS-10PSEO, respectively. The incorporation of SS and PSEO into the G–Ch matrix resulted in a reduction in air permeability compared to the pristine sample. This behavior can be attributed to the formation of more tortuous pathways for gas diffusion within the fibrous network, which restricts airflow. As a result, the modified nanofibrous mats exhibit improved air barrier properties. These characteristics make G–Ch–SS and G–Ch–SS-10PSEO promising candidates for food-packaging applications where controlled gas exchange is required.

The tensile strength of G-Ch nanofibrous mats was 0.72 ± 0.15 MPa, while the incorporation of SS slightly increased the value to 0.78 ± 0.18 MPa. This slight improvement may be attributed to the reinforcing effect of SS within the gelatin/chitosan matrix. Polysaccharide and protein blending can enhance the mechanical properties of nanofibers by improving tensile strength, elasticity, and thermal stability compared to single-component fibers [26]. However, the addition of 10 wt.% PSEO decreased the tensile strength to 0.56 ± 0.16 MPa and 95.40 ± 17.0495. 40 ± 17.04 reduced the elongation at break to 7.95 ± 2.8%. This reduction suggests that PSEO may weaken the intermolecular interactions between gelatin, chitosan, and SS, disturb the fiber network integrity, and increase the brittleness of the nanofibrous mats.

The swelling ratio and moisture uptake values of the nanofibrous mats are presented in Table 4. The G–Ch nanofibrous mats showed a swelling ratio of 312.4 ± 18.6%, indicating the high-water affinity of the gelatin/chitosan matrix. This behavior can be attributed to the abundance of hydrophilic functional groups such as hydroxyl, amino, and amide groups in gelatin and chitosan, which can interact with water molecules through hydrogen bonding. After the incorporation of snail slime, the swelling ratio increased to 356.8 ± 21.7%. This increase may be related to the hydrophilic proteinaceous, glycoprotein, and polysaccharide fractions present in snail slime, which can enhance water absorption within the fibrous matrix. In contrast, the G–Ch–SS–10PSEO mats exhibited a lower swelling ratio of 238.5 ± 15.3%. The reduction in swelling after PSEO incorporation can be explained by the hydrophobic nature of the essential oil and the possible occupation of free volume within the gelatin–chitosan–snail slime network by hydrophobic terpene compounds. In addition, the increased fiber diameter and reduced air permeability observed for the PSEO-containing mats suggest the formation of a more compact fibrous structure, which may restrict water penetration into the mat. A similar trend was observed for moisture uptake. The moisture uptake values were 24.8 ± 1.6%, 29.6 ± 1.9%, and 18.7 ± 1.3% for G–Ch, G–Ch–SS, and G–Ch–SS–10PSEO, respectively. The higher moisture uptake of G–Ch–SS confirms the contribution of hydrophilic snail slime components, whereas the lower value of G–Ch–SS–10PSEO indicates improved resistance to moisture absorption. These findings are consistent with the water contact angle results, where PSEO incorporation increased the surface hydrophobicity of the mats.

### 3.5. PSEO Retention After Thermal Treatment

Since PSEO is composed mainly of volatile terpene compounds, the possible loss of essential oil during thermal treatment was evaluated spectrophotometrically. The PSEO content of the untreated G–Ch–SS–10PSEO nanofibrous mat was 84.7 ± 2.8 mg PSEO equivalent/g mat. After thermal treatment at 120 °C for 2 h, the remaining PSEO content decreased to 58.9 ± 3.2 mg PSEO equivalent/g mat, corresponding to a retention of 69.5 ± 4.1%. This result confirms that thermal treatment caused partial loss of volatile PSEO constituents. However, a considerable amount of PSEO was retained within the nanofibrous structure, most likely due to physical entrapment within the gelatin–chitosan–snail slime matrix and interactions between the hydrophobic essential oil components and the polymer network.

The retained PSEO fraction is consistent with the functional performance of the thermally treated mats. Despite partial volatilization during heating, the G–Ch–SS–10PSEO formulation showed the highest antioxidant and antibacterial activities and the most effective preservation performance in chicken wings during refrigerated storage. Therefore, the results suggest that the thermal treatment provided structural stabilization of the nanofibrous mats while maintaining a sufficient amount of bioactive PSEO to support active packaging functionality.

### 3.6. Antioxidant Activity

The antioxidant activity of the G–Ch, G–Ch–SS, and PSEO-incorporated nanofibrous mats was evaluated using DPPH and ABTS radical scavenging assays (Table 5). The G–Ch nanofibers exhibited limited radical scavenging ability, with inhibition values below 14% for both assays. This can be attributed to the absence of significant bioactive compounds capable of donating hydrogen atoms or electrons. The incorporation of SS resulted in a slight increase in antioxidant activity, reaching 15.6 ± 1.0% (DPPH) and 20.4 ± 1.2% (ABTS). This enhancement can be attributed to the presence of bioactive constituents such as glycoproteins, uronic acids, and sulfated polysaccharides that possess mild hydrogen- donating and metal-chelating abilities [27,28]. A pronounced increase in antioxidant activity was achieved by incorporating 10 wt% of PSEO into the G–Ch–SS matrix, where DPPH and ABTS scavenging activities reached 36.8 ± 2.0% and 42.7 ± 2.2%, respectively. This enhancement is primarily linked to the rich monoterpene and sesquiterpene profile of PSEO, particularly 3-carene, α-pinene, myrcene, β-pinene, camphene, caryophyllene, α-terpinolene, and α-cadinene, which were identified in the GC–MS analysis. This enhancement is primarily linked to the rich monoterpene and sesquiterpene profile of PSEO, particularly α-pinene, β-pinene, limonene, camphene, and bornyl acetate. Those molecules are recognized as strong electron donors capable of quenching both DPPH• and ABTS•^+^ radicals through hydrogen-atom transfer and single-electron transfer mechanisms [29,30]. The higher activity observed in the ABTS assay may be related to the increased electron -transfer capability of these compounds in more polar environments [31]. In addition, the combined presence of SS and PSEO may contribute to the overall antioxidant performance through complementary effects. The presence of mucopolysaccharides in SS may also contribute to the stabilization of bioactive compounds within the nanofibrous matrix, as suggested in previous studies [32]. Furthermore, functional groups in gelatin and chitosan may participate in weak interactions with terpenoid radicals, contributing to the overall antioxidant performance [33]. The progressive increase in antioxidant activity from G-Ch to G–Ch–SS-10PSEO indicates that the antioxidant performance of the nanofibers is largely governed by the content and accessibility of bioactive terpenes. Similar trends have been reported in essential oil-loaded biopolymer nanofibers, where increasing oil content resulted in enhanced radical scavenging activity [34].

### 3.7. Antibacterial Activity

The antibacterial activity of the nanofibrous mats was evaluated against *E. coli* and *S. aureus*, and the results are presented in Table 5. The G–Ch formulation exhibited negligible antibacterial effect, with inhibition zones below 2 mm, reflecting the limited intrinsic antimicrobial capability of the base polymer matrix. The addition of SS (G–Ch–SS) resulted in a moderate improvement, with inhibition zones of 2.5 ± 0.5 mm for *E. coli* and 4.1 ± 0.6 mm for *S. aureus*. This enhancement is attributed to the presence of glycoproteins, polycationic peptides, and sulfated mucopolysaccharides in SS that can disrupt microbial adhesion and interfere with membrane integrity [35]. A significant increase in antibacterial efficacy was observed for the G–Ch–SS–10PSEO mats, which generated inhibition zones of 0.3 ± 0.6 mm for *E. coli* and 10.2 ± 0.7 mm for *S. aureus*. The marked improvement is associated with the terpene-rich composition of PSEO, particularly 3-carene, α-pinene, myrcene, β-pinene, camphene, caryophyllene, and α-terpinolene, which may contribute to antibacterial activity through disruption of membrane integrity and interference with microbial metabolic processes. [36]. Monoterpenes are known to interact with bacterial members and may disrupt cellular integrity, contributing to the observed antimicrobial activity in the nanofibrous mats [37].

*S. aureus* showed higher sensitivity compared *to E. coli*, which is consistent with the structural differences between Gram-negative bacteria. The outer membrane of Gram-negative bacteria acts as a barrier to hydrophobic compounds, whereas Gram-positive bacteria are generally more susceptible to such agents. Similar Gram-dependent responses were reported by Akhouy et al. (2024a) [8] for chitosan-based nanofibers containing *Origanum elongatum essential oil* and by Mendes et al. (2023) [38] for *peppermint* oil-loaded PLA nanofibers, supporting the higher susceptibility of Gram-positive bacteria to EO-rich systems.

The progressive improvement from G-Ch to G–Ch–SS-10PSEO suggests a combined antibacterial contribution of SS and PSEO. While SS provides additional bioactive functionality, the essential oil serves as the primary source of antimicrobial activity. The presence of both components may contribute to enhanced interactions with bacterial cells, leading to improved antibacterial performance.

### 3.8. Food Preservation Performance

**Figure 7:**
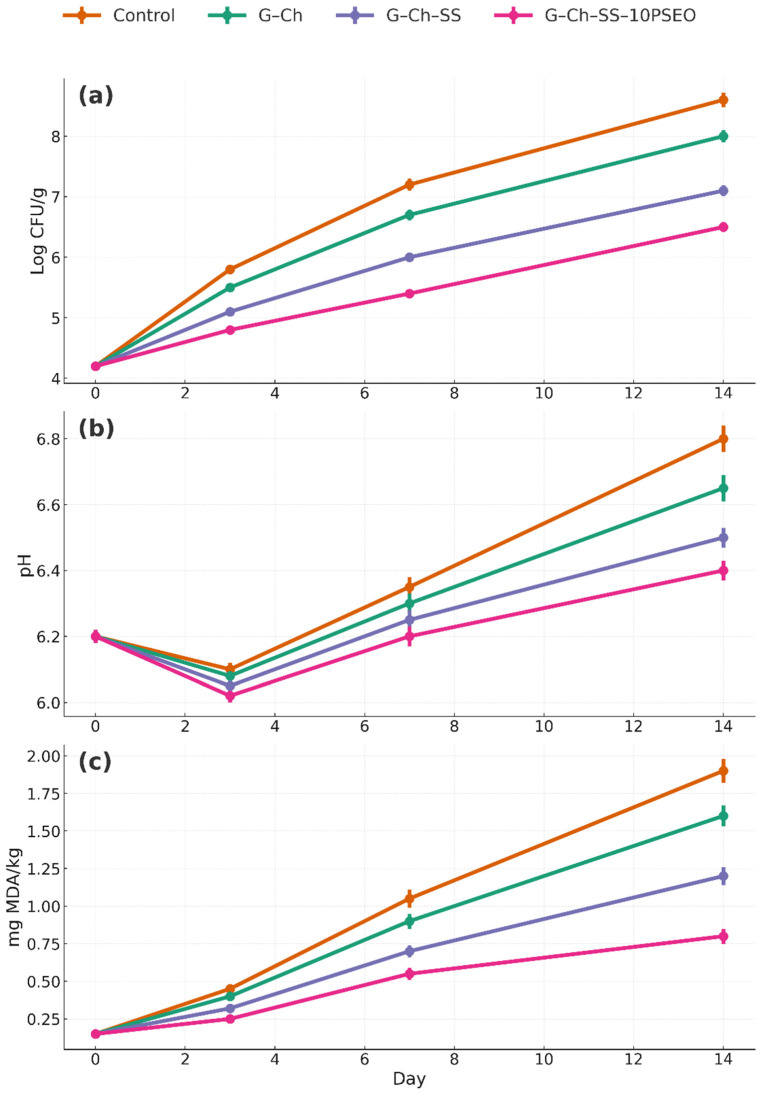
Changes in (**a**) total viable counts (TVC), (**b**) pH, and (**c**) thiobarbituric acid reactive substances (TBARS) of chicken wings wrapped with different nanofibrous mats.

The evolution of microbial load, pH, and lipid oxidation in chicken wings wrapped with different nanofibrous mats during refrigerated storage is shown in Figure 7, where Figure 7a illustrates the changes in total viable counts (TVC), Figure 7b presents the pH evolution, and Figure 7c depicts the progression of lipid oxidation based on TBARS values.

As shown in Figure 7a, the control group exhibited the most rapid deterioration, with TVC reaching 8.60 log CFU/g by day 14, reflecting the high perishability of poultry meat. In contrast, all nanofiber-treated samples reduced microbial proliferation, with the strongest inhibition observed for G–Ch–SS–10PSEO (~6.50 log CFU/g), followed by G–Ch–SS and G-Ch. This trend is consistent with the antibacterial performance observed in Section 3.6 and aligns with previous studies [39,40]. SS-containing nanofibers (G–Ch–SS) demonstrated intermediate inhibition, which can be attributed to the presence of bioactive glycoproteins and peptides that may interfere with bacterial adhesion and metabolic activity [41].

The pH evolution (Figure 7b) followed a similar pattern. An initial decrease was observed during early storage, followed by a gradual proteolysis and accumulation of alkaline nitrogenous compounds during Spoilage. In contrast, G–Ch–SS-10PSEO maintained the lowest value (~6.4), indicating reduced microbial and enzymatic activity. As shown in Figure 7c, lipid oxidation increased progressively during storage, with the control sample reaching the highest TBARS value (~1.9 mg MDA/kg). In contrast, G–Ch–SS-10PSEO exhibited significantly lower oxidation (~0.80 mg MDA/kg), suggesting the contribution of antioxidant components within the nanofibrous matrix [42]. Overall, the incorporation of SS and PSEO into the nanofibrous mats improved preservation performance by reducing microbial growth, limiting pH increase, and suppressing lipid oxidation, indicating their potential for extending the shelf life of perishable food products.

## 4. Conclusions

Biodegradable gelatin–chitosan nanofibrous mats functionalized with snail slime (SS) and *Pinus sylvestris* essential oil (PSEO) were successfully produced via solution blow spinning and evaluated as active food-packaging materials. The incorporation of SS and PSEO influenced the structural and surface properties of the nanofibers, leading to increased fiber diameter, enhanced hydrophobicity and reduced air permeability. Spectroscopic and structural analyses indicated that the active components were incorporated without significant chemical modification of the polymer matrix. The addition of SS and PSEO improved the antioxidant performance, with the highest activity observed for the G–Ch–SS-10PSEO formulation. Similarly, antibacterial activity was significantly enhanced, with the same formulation showing the strongest inhibition against *E. coli* and *S. aureus*. Application tests on chicken wings demonstrated that the developed nanofibrous mats effectively reduced microbial growth, limited pH increases, and suppressed lipid oxidation during 14 days of refrigerated storage. Overall, these findings highlight the potential of SS- and PSEO-functionalized nanofibrous mats as promising candidates for sustainable and effective active food-packaging applications.

Despite the promising results obtained in this study, some limitations should be acknowledged. The physicochemical properties of the spinning solutions, including viscosity, conductivity, and surface tension, were not systematically characterized, which limits a deeper understanding of the fiber formation mechanisms. In addition, important barrier-related parameters such as the water vapor transmission rate, oxygen permeability, and swelling behavior were not evaluated. Future studies should focus on comprehensive solution characterization, optimization of the PSEO loading concentration, detailed moisture and gas barrier analyses, and long-term food preservation performance to further validate the practical applicability and scalability of these nanofibrous mats in active food-packaging systems.

## Figures and Tables

**Table 1:** Composition and preparation conditions of polymer formulations.

Solution Code	Polymer/ Active Composition	Total Concentration	Solvent System	Preparation Conditions	Additional Active Components
G-Ch	Gelatin/chitosan (4:1 *w*/*w*)	14 wt%	Acetic acid/Formic acid (60:40 *w*/*w*)	Gelatin dissolved at 70 °C under magnetic stirring for 3 h, followed by chitosan addition and continuous stirring for 5 h to ensure complete dissolution and homogeneous polymer interaction	-
G–Ch–SS	Gelatin/chitosan/Snail slime powder	14 wt%	Acetic acid/Formic acid (60:40 *w*/*w*)	Snail slime powder incorporated into the G-Ch solution and stirred at 60 °C for 4 h to obtain homogeneous solution	5 wt.% snail slime powder (relative to polymer weight)
G–Ch–SS–10PSEO	Gelatin/chitosan/Snail slime powder/PSEO	14 wt%	Acetic acid/Formic acid (60:40 *w*/*w*)	PSEO added to G–Ch–SS solution and mixed for 30 min	10 wt% PSEO (relative to polymer weight)

**Table 2:** Composition of PSEO.

N°	Rt	Identified Compounds	Mol Formula	% Area
1	12.877	α-Pinene	C_10_H_16_	20.11
2	8.234	Myrcene	C_10_H_16_	10.7
3	22.822	camphene	C_10_H_16_	4.3
4	12.515	β-Pinene	C_10_H_16_	8.7
5	9.655	Sabinene	C_10_H_16_	2.5
6	13.182	3-Carene	C_10_H_16_	30.14
7	11.995	α-Limonene	C_10_H_16_	2.1
8	7.468	p-cymene	C_10_H_14_	1.3
9	13.119	α-terpinolene	C_10_H_16_	2.7
10	8.659	Terpinen-4-ol	C_10_H_18_O	1.2
11	14.009	trans-Pinocarveol	C_10_H_16_O	0.4
12	14.511	Verbenone	C_10_H_14_O	0.3
13	12.154	Caryophyllene	C_15_H_24_	8.9
14	9.097	α-Humulene	C_15_H_24_	1.9
15	14.303	α-Murolene	C_15_H_24_	0.8
16	15.329	α-Cadinene	C_15_H_24_	3.7
		Identified from the total area		99.75

**Table 3:** Composition of SS.

Organic Compound	Quantity	Unity
Allantoin	6.05	mg/g
Glycolic Acid	8.15	mg/g
Collagen	489.5	mg/g
Polyphenols	105.12	mg/g
Vitamin B1	0.32	mg/g
Vitamin A	0.11	u.i/kg
Vitamin E	0.25	u.i/kg
Vitamin C	2.58	u.i/kg
Peptide Cardiovascular	12.58	%
Protein	2.03	g/100 g
Amino Acid	1.231	g/100 g
Polysaccharide	1.65	g/100 g
Flavonoids	56.12	mg/g
Minerals	Quantity	Unity
Calcium	0.05	mg/kg
Magnesium	8	mg/kg
Iron	0.01	mg/kg
Potassium	6	mg/kg
Zinc	10	mg/kg

**Table 4:** Physicochemical, barrier, mechanical, and water-sensitivity properties of the nanofibrous mats.

Sample Code	Contact Angle (°)	Air Permeability (mm/S)	Max Strength (MPa)	Max Strain (%)	Swelling Ratio (%)	Moisture Uptake (%)
G-Ch	86.56 ± 11.60	20.66 ± 0.58	0.72 ± 0.15	14.44 ± 4.4	312.4 ± 18.6	24.8 ± 1.6
G–Ch–SS	85.47 ± 3.22	13 ± 0	0.78 ± 0.18	10.43 ± 3.8	356.8 ± 21.7	29.6 ± 1.9
G–Ch–SS-10PSEO	95.40 ± 17.04	14.66 ± 0.58	0.56 ± 0.16	7.95 ± 2.8	238.5 ± 15.3	18.7 ± 1.3

**Table 5:** Antioxidant and antimicrobial activity of nanofibers.

Samples	DPPH (%)	ABTS (%)	*E. coli* (mm)	*S. aureus* (mm)
G-Ch	10.2 ± 0.7 ^c^	13.8 ± 0.9 ^c^	0.8 ± 0.4 ^c^	1.7 ± 0.5 ^c^
G–Ch–SS	15.6 ± 1.0 ^b^	20.4 ± 1.2 ^b^	2.5 ± 0.5 ^b^	4.1 ± 0.6 ^b^
G–Ch–SS-10PSEO	36.8 ± 2.0 ^a^	42.7 ± 2.2 ^a^	7.3 ± 0.6 ^a^	10.2 ± 0.7 ^a^

Different superscript letters (a–c) within the same column denote statistically significant differences (*p* < 0.05).

## Data Availability

The original contributions presented in this study are included in the article. Further inquiries can be directed to the corresponding authors.
